# Knowledge Attitude and Practices of Mitanin's (Community Health Workers) in Chhattisgarh: Malaria Elimination Perspective

**DOI:** 10.3389/fpubh.2021.774864

**Published:** 2022-03-03

**Authors:** Raju Ranjha, Chander Prakash Yadav, Mehul Kumar Chourasia, Nitika, Chinmay Kumar Dash, Jitendra Kumar

**Affiliations:** ^1^Field Unit, ICMR-National Institute of Malaria Research, Raipur, India; ^2^ICMR-National Institute of Malaria Research, New Delhi, India; ^3^Division of Population Health and Genomics, School of Medicine, University of Dundee, Dundee, United Kingdom; ^4^National Vector Borne Disease Control Programme, Raipur, India

**Keywords:** community health workers (CHWs), malaria, knowledge attitude and practice (KAP), tribals, malaria endemic areas

## Abstract

**Background:**

For the success of any program, its implementation plays a crucial role. Community health workers are of immense importance for malaria elimination from India.

**Objective:**

This study was aimed to assess the knowledge gaps and the responsible factors for mitanins' knowledge on various aspects of and problems faced by mitanins during their work.

**Methods:**

Structured interviewer-based questionnaire was used to collect the data, and ordinal regression was applied to analyze the data.

**Results:**

Only 26% of the mitanins were having a good knowledge attitude and practices (KAP) score about malaria. Malaria endemicity of area [odds ratio (OR) = 0.26, 95% CI = 0.13–0.50), *P* < 0.001] and education (OR = 0.35, 95% CI = 0.18–0.69, *P* = 0.002) were the two significant factors affecting the KAP of mitanins.

**Conclusion:**

This study shows that prioritizing education while recruiting the mitanins and training them in the low endemic areas with a focus on malaria, which will help achieve the malaria elimination goal.

## Introduction

India has a target of eliminating malaria by 2030 (1). Malaria control and elimination in rural tribal areas are one of the roadblocks in India's malaria elimination drive (2). The problems in the health sector in tribal areas are compounded by difficult-to-reach areas and poor access to health facilities (3). Several tools utilized for malaria control in India are Long-Lasting Insecticidal Nets (LLINs) and Rapid Diagnostic Tests (RDTs) (2). Although these tools and techniques are of immense importance for malaria elimination, the correct implementation of those tools is the key (4). Due to the huge shortfall of physicians and nurses in the rural and tribal areas, the community health workers (3) (CHW) become the key players for implementing any program.

The malaria elimination program is dependent on LLINs, medicines, and RDTs. These are very good tools for achieving short-term goals. But the pace of malaria elimination in India will depend highly on the skills and knowledge of CWHs. CHWs are an important human resource and contribute significantly to malaria control (5). CHWs are trusted members of the community with a very good understanding of the community (6). They are trusted in the community owing to the same language, ethnicity, and socioeconomic status. The CHWs act as a liaison between healthcare providers and minority/poor communities in rural areas, thus adding value to the healthcare teams. The CHW program in Chhattisgarh is called Mitanin Program. Mitanin in Chhattisgarhi language means friend. The mitanin program was launched in Chhattisgarh in 2002, with the broad objective of providing immediate relief from common health problems and improving health awareness in the rural areas of Chhattisgarh. The village community selects the mitanin in hamlet level meetings and her selection is approved by the village panchayat (Local self govt. body). Currently, there are nearly 60,000 functional mitanins in the state. All the mitanins are supplied with a drug kit which is refilled regularly (7). Mitanins are trained for the community control of malaria and are provided with a guidebook for malaria control and treatment. They act as drug depots to provide the medicines to the patients, carry out the Rapid diagnostic test, and collect blood slides for malaria diagnosis (7).

Chhattisgarh is one of the malaria-endemic states in India. It is inhabited by 2.3% of India's population, but it contributed significantly to malaria morbidity and mortality in India in 2019, 17.8 and 40.3%, respectively (8). The API of the state was 1.97 in 2019. A total of 11 out of 27 districts had an annual parasite index (API) of more than one with the highest API of 44.31 in the Bijapur district (8). More than 30% of the Chhattisgarh population is tribal and lives in forested areas (9). The tribal communities have poor health indicators compared to others. Tribal communities are the most difficult to test and treat (10), thus, increasing the importance of CHWs in these areas. Women and children, being the most vulnerable, are severely affected by different illnesses, and also by lack of awareness on malaria, transportation, discriminatory behavior by healthcare providers, and financial constraints that make CHW of prime importance for delivering health services to them (11). Mitanins being the important and grass root level worker in the health system, their knowledge and skills are important for the effective implementation of health programs. Gaining social recognition, a sense of social responsibility, and self-efficacy motivates the CHWs (12). It is of immense importance from the standpoint of malaria elimination in the country. This study aimed to find out the knowledge gaps and skills of Mitanins in Chhattisgarh and find out the factors that may help in improving the performance of Mitanins for malaria control and elimination in the area.

## Materials and Methods

### Study Area and Study Participants

**Figure 1:**
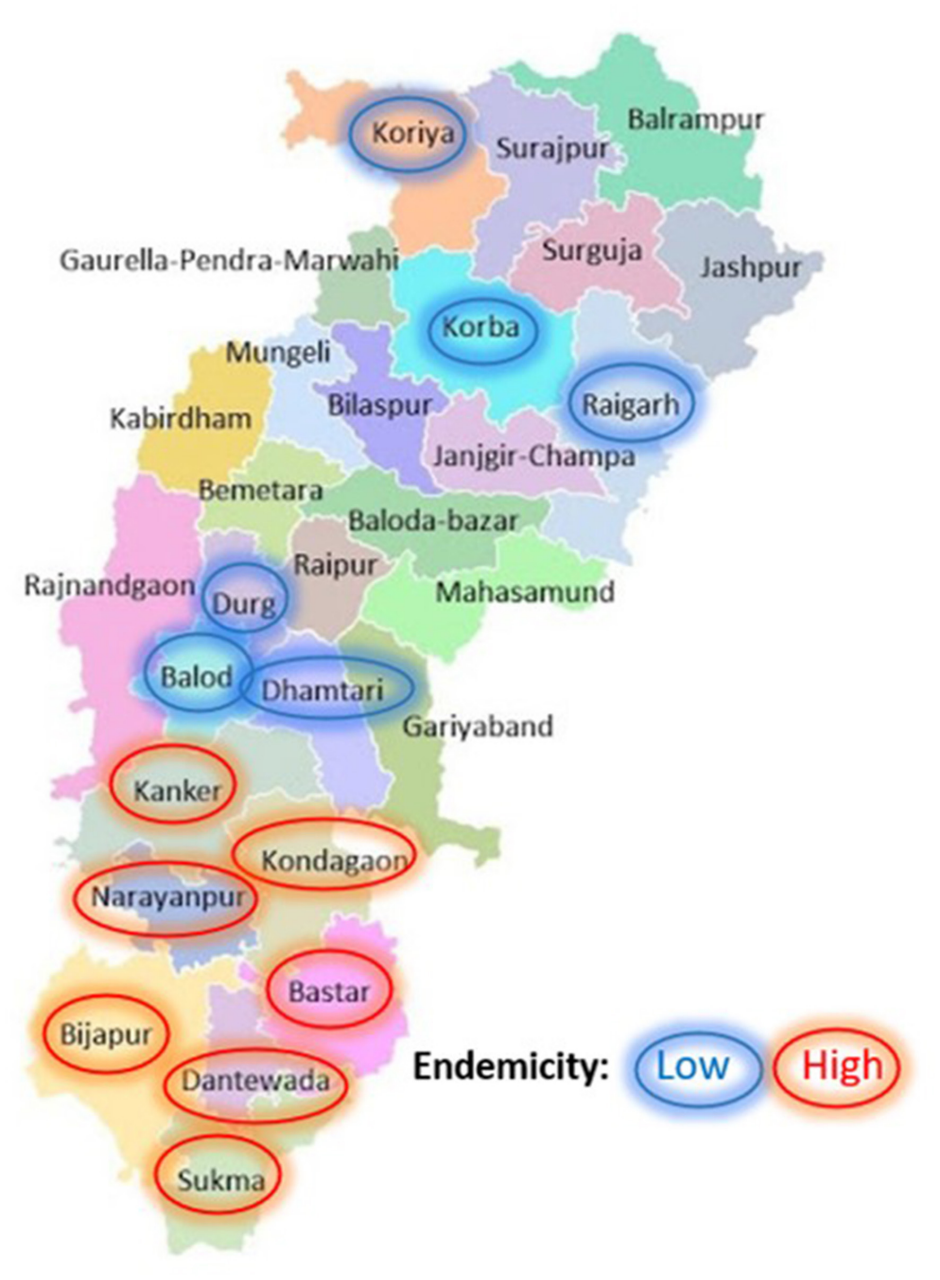
Distribution of selected study districts and their endemicity. The study included mitanin from 13 districts, 6 low endemic (circled blue) and 7 high endemic (circled red) were included in the study.

Chhattisgarh is geographically plain with topographic variations, including plain, foothill, and forested and non-forested areas. This was a cross-sectional study carried out in high malaria endemic as well as low endemic districts of Chhattisgarh. Two subcenter in each district were selected and five villages from the two subcenters were included in the study. An approximate population in each subcenter is 3,000–5,000. There is one mitanin for ~250 population. The sample size was calculated for the total mitanins in the selected subcenters. It came out to be 200 at a 95% CI and a 5% margin of error. This study was approved by the Institutional Ethics committee ICMR-National Institute of Malaria Research, New Delhi, IEC no- ECR/NIMR/EC/2018/211. Written consent was obtained from the participants in the study. 203 Mitanins were included in this study from April 2018 to December 2019. This study was carried out in 68 villages of 13 districts of Chhattisgarh. The study districts were divided into two groups: low endemic and high endemic (Figure 1).

### Data Collection and Grading

A structured questionnaire was used to collect information from the mitanins. A pre-tested questionnaire from earlier studies was used for this purpose (13). It was standardized for forested and malaria-endemic tribal regions. Two staff members were trained for the study. The study questionnaire was divided into six sections. The first two sections were regarding the socio-demographics and malaria training-related information. The third and fourth sections were to find out their knowledge about malaria diagnosis and treatment, respectively. The fifth section was focused on their knowledge about malaria prevention with a focus on LLINs. In the last section, documentation by the Mitanins was assessed. The five sections were graded equally, allotting five marks for each section. The total KAP score was calculated out of a maximum of 25 grades. Mitanin knowledge was graded poor, average, and good for <40, 40–70, and >70% grades.

### Data Analysis

All the study data were entered in Epi Info (CDC, Atlanta, Georgia, US). The cleaned data were analyzed using the Statistical Package for the Social Sciences (SPSS) version 20 (IBM Corp, Armonk, NY, USA). All categorical variables were reported as frequency (percentages), and continuous variables were reported as means and SD. Univariate and multivariate ordinal regression analysis was used, and mitanins' KAP score was the dependent variable in the analysis. The mitanins' age, education, work experience, and area endemicity for malaria were used as independent variables. The mitanins' education and experience were dichotomized. Assumptions for ordinal regression were checked for assessing the validity of the regression model. A *P*-value < 0.05 was considered significant for all the analyses.

## Results

### Baseline Characteristics and Training Details

A total of 203 Mitanins with a nearly 1:1 ratio based on endemicity were included in the study (Figure 1). About half (53%) of the participants were educated until primary level or above. The mean age of the participants was 38.54 (9.31), and the average experience was 11.51 (5.61) years.

All the mitanins were trained, and more than two-thirds of the Mitanin's were willing to take a refresher training on malaria. About 19% of Mitanins felt that the training batches were overcrowded, and about 13% faced language issues. Baseline characteristics and training details are illustrated in Table 1.

**Table 1:** Baseline characteristics and training status of the study population (*n* = 203).

**Variable**	**Category**	***n* (%)**
Serving area (%)	High endemic	108 (53.2)
Age (in years)		38.54 (9.31)
Education (in years)		5.24 (4.21)
Experience (in years)		11.51 (5.61)
Education (categorized) (*n* = 203)	Less than primary	82 (40.4)
	Primary and above	121 (59.6)
Experience (categorized) (*n* = 198)	Less than 9 years	70 (35.4)
	9–16 years	76 (38.4)
	17 years and above	52 (26.3)
**Training status**	
Received malaria training	Yes	203 (100)
Regular training on malaria	Yes	185 (92)
Average mitanin per session		26.32 (8.1)
Overcrowded	Yes	38 (18.8)
Training languages	Local	203 (100)
Language problem	Yes	27 (13.4)
Use of different teaching aids	Yes	196 (97)
Content of training	Appropriate	191 (94.1)
Refresher training required	Yes	179 (88.2)

*Age, experience, education and average mitanins per session expressed as mean (SD)*.

### Mitanins KAP Performance

**Figure 2:**
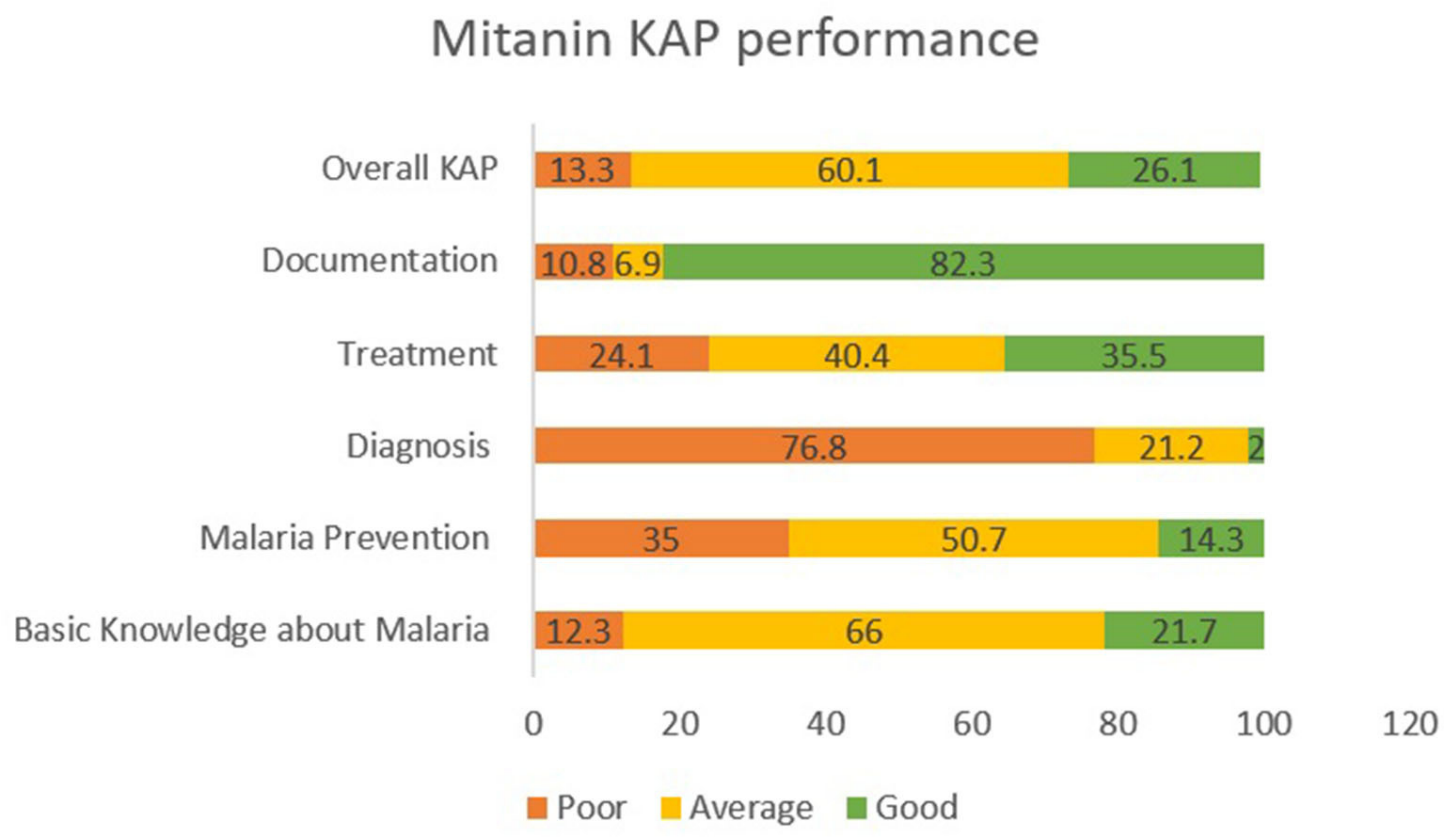
Mitanin KAP Scores. The KAP score of mitanins in different sections and overall scores are highlighted in green (Good), Yellow (Average) and Orage (Poor). Malaria diagnosis, prevention and treatment were the three sections where frequency of low scoring was high.

About 60% of mitanins had an average KAP score, about 26% had good KAP scores, and 13.3% had poor KAP scores. For malaria, diagnosis, prevention, and treatment section, the frequency of mitanins with the low scores was high, 76.8, 35, and 24.1%, respectively. Documentation and basic malaria were the sections where the mitanins scores were high (Figure 2).

### Determinants Affecting Mitanins' Performance

Table 2 shows the results of ordinal logistic regression analysis. Endemicity and education were the two variables affecting the mitanins' KAP score significantly. The proportional odds of higher KAP were 0.26 (95% CI = 0.13–0.50), *P* < 0.001, and 0.35 (95% CI = 0.18–0.69), *P* = 0.002 in the low endemic areas and mitanins with an education level less than primary compared to high endemic areas and mitanins with higher education level respectively. Education was the factor affecting most of the components of mitanins' KAP. The section-wise results of the regression analysis of the mitanin KAP analysis are given in Supplementary Tables 1–3.

**Table 2:** Factors affecting mitanin's performance using ordinal logistic regression (*n* = 203).

**Variable**	**Category**	**Univariate analysis**		**Multivariate analysis**	
		**cOR (95%CI)**	***P*-value**	**aOR (95%CI)**	***P*-value**
Endemicity	Low	0.33 (0.18–0.59)	**<0.001**	0.26 (0.13–0.50)	**<0.001**
	High	1		1	
Age	In years	0.97 (0.94–1.002)	0.069	0.99 (0.96–1.03)	0.829
Education	Less than 5 years	0.46 (0.26–0.82)	**0.008**	0.35 (0.18–0.69)	**0.002**
	5 years and more	1		1	
Experience	Less than 9 years	2.06 (1.002–4.25)	**0.049**	1.01 (0.41–2.5)	0.97
	9–16 years	2.91 (1.4–6.00)	**0.004**	1.58 (0.72–3.49)	0.258
	17 years and more	1		1	

*P-value < 0.05 statistically significant*.

*Likelihood ratio Chi-square 29.9; <0.001 Pseudo R^2^ – 0.16*.

*Statistically significant values are shown in bold*.

### Problems Faced by Mitanins

**Figure 3:**
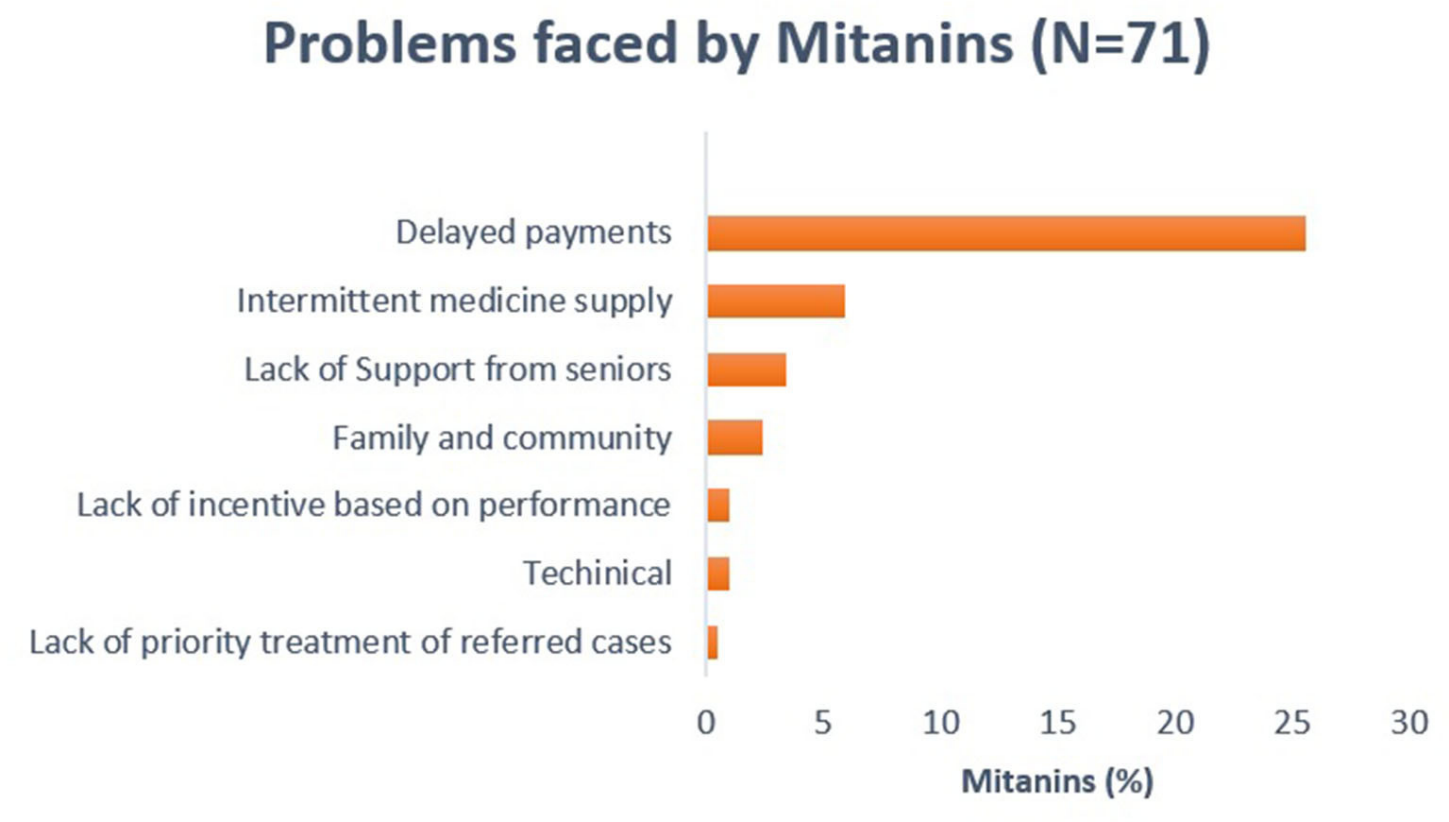
Problems faced by mitanins. Delayed payments and intermittent medicine supply are major problems faced by mitanins.

About 35% of the mitanins faced problems related to their work; delay in payments and intermittent medicine supply were the two major problems (Figure 3). While lack of support from seniors or family members were other common problems reported by the mitanins.

## Discussion

Good knowledge of malaria control interventions will ensure better performance of CHWs (14). This study was planned to find out the knowledge of mitanins about malaria control in Chhattisgarh and identify the important factors that may be crucial for improving the knowledge of mitanins.

The mitanins' KAP score was affected by their education [odds ratio (OR) = 0.35, 95% CI = 0.18–0.69, *P* = 0.002] and endemicity [(OR = 0.26, 95% CI = 0.13–0.50), *P* < 0.001] of the area being served by the mitanin (Table 2).

In low-endemic areas, the odds of high KAP were low. Accredited Social Health Activists (ASHA) in the low-endemic areas had low knowledge about vector breeding, malaria symptoms, and diagnosis compared to counterparts in high endemic areas in northeast India (15). The low knowledge of malaria of mitanins in the low-endemic areas indicates changing focus due to low malaria cases. In the WHO guidelines, surveillance is the core intervention for malaria elimination when the cases go very low (16). The knowledgeable community health workers are of immense importance for making surveillance a core intervention for malaria elimination. Training, monitoring, and assessing mitanins lead to significant improvement in their knowledge from the malaria elimination perspective (17). Separate training sessions on malaria may help fill the knowledge gap of mitanins in the low-endemic areas.

The proportional odds of having a higher KAP were low in the mitanins with low-level education. As per the National Health Mission (NHM), India guidelines for ASHA, a formal level of education up to the 8th class is the criteria for a mitanin's recruitment (18). In our study, only about 19% of the mitanins had an education of 8th class or above. The level of education was reported to be the critical determinant for knowledge of disease and its transmission for individuals involved in integrating community efforts for dengue control (19). Our analysis found that education was the only factor affecting the mitanin's basic knowledge about malaria (OR = 0.39, 95% CI = 0.20–0.80, *P* = 0.01) (Supplementary Table 1). The higher education level of CWHs was reported to be associated with good record-keeping, better use of aids, and better counseling (20). Mousoke et al. (21) suggested that the level of education must be given priority while recruiting CHWs. Improving literacy in the tribal areas will positively impact malaria control in the area (22). Our study supports the earlier observation that mitanins with higher education levels have better KAP. The non-availability of educated individuals may be a factor affecting mitanins recruitment in rural and tribal areas. Also, giving education a priority while recruiting the mitanins may help in the success of the malaria elimination program.

Malaria diagnosis, prevention, and treatment were the three areas where the frequency of mitanins with below-average knowledge was high, 76.8, 35, and 24.1%, respectively. Chowdhury et al. (15) reported a significant difference in the ability to perform by ASHAs in low-endemic and high-endemic areas. 57.24 and 83.16% of ASHAs in the ASAHs in low-endemic and high-endemic areas were able to perform RDT (15). In our study, about 71% of the mitanins correctly told the steps for conducting RDT, but only about 9% could tell the correct time for reading the results but there was no significant difference with the endemicity of the area being served. The low performance in the diagnosis was due to the discrepancy in the RDT protocol and the protocol of the kits provided to the mitanins. Updating mitanins, whenever the kit changed with a new kit having a different protocol, may help in improving the malaria diagnosis by the mitanins.

Mitanins' knowledge about malaria prevention is affected by the endemicity of the area being served. Mitanins of low endemic areas had lower KAP about malaria prevention compared to the mitanins of low endemic areas (OR 0.31, 95% CI 0.17–0.60; *P* < 0.001) (Supplementary Table 2). The main malaria prevention tools LLINs and IRS are applied only in the high endemic areas (23). Involvement in the ongoing malaria prevention activities in the area may be the reason for high KAP about malaria prevention in the high endemic areas compared to low-endemic areas.

Mitanins' knowledge about malaria treatment is being affected by all the variables considered, including endemicity, education, age, and experience of mitanin (Supplementary Table 3). Higher KAP rates about malaria treatment were high in mitanins serving high endemic areas, having higher education and lower age. The low knowledge about malaria treatment in mitanins of low-endemic areas may be due to their dependence on ANM for the same (15). Our study supports the earlier observation that ASHAs in the high endemic areas have significantly better knowledge about malaria treatment compared to low-endemic areas (15). The mitanins with 9–16 years of experience had higher odds of having better KAP about malaria treatment (OR = 22.43, 95% CI = 1.15–5.12; *P* = 0.01). The higher experience of health workers was found to be correlated with better knowledge (24). In our regression analysis, the experience was found to be interacting with education. The frequency of mitanins with primary education and above was higher (~44%) in the experience level of 9–16 years compared to 17 years and above and <9 years of experience, 23 and 34% respectively. This may be the reason for lower knowledge about malaria treatment in mitanins with experience 17 years and above.

The sustained success of the malaria control and elimination program depends on the data analysis and implementation based on the data (25). Malaria elimination in India will require high-quality real-time data for decision-making, as well as data collection and documentation. The mitanins' performance was good in this section, with 82% of the mitanins having a good score. Mitanins with education level primary and above had good KAP, although it was not significant (OR = 0.43, 95% CI = 0.18–1.04, *P* = 0.06).

Delay in payments and intermittent medicine supply are the two key problems faced by mitanins in the area. Lack of continuous medicine supply may hinder the mitanin from performing their duties effectively. Timely payments are required to keep the mitanins motivated. Smaller, irregular, and delayed payments were problems highlighted in the earlier studies (13, 26). ASHAs/Mitanins were willing to take new responsibilities but expect more incentives. Due to low incentives, they look for other paid jobs along with their healthcare services (27). Getting timely payments may help motivate mitanins for community services.

In conclusion, our study shows that mitanins' KAP performance is affected by the endemicity of the area being served and their education level. The changing focus in low-endemic areas may become a hurdle for bringing malaria cases to zero in those areas. Considering the education of individuals while engaging in the mitanin program may be highly helpful for the long-term goal of malaria elimination. Social and behavioral aspects affecting the utilization of malaria control services in the community can be improved by having a knowledgeable mitanin in the area. Mitanin malaria training should focus on removing the knowledge gap in diagnosing, preventing, and treating malaria. Mitanins in the low-endemic areas needs to be trained more on malaria. Timely payments are required to keep the mitanins motivated. Despite having good diagnostics and effective medicines, there are deaths due to malaria. In 2020, 63 deaths due to malaria were reported in India. Most of the malaria cases and deaths in Chhattisgarh are from rural areas. Mitanins with good knowledge about malaria control may effectively reduce mortality to zero much before malaria elimination from the country.

## Data Availability Statement

The original contributions presented in the study are included in the article/Supplementary Material, further inquiries can be directed to the corresponding author.

## Ethics Statement

The studies involving human participants were reviewed and approved by Institution Ethics Committee, ICMR-National Institute of Malaria Research, New Delhi. The patients/participants provided their written informed consent to participate in this study.

## Author Contributions

RR conceptualized the study and wrote the manuscript. RR and CY designed the study. RR, CY, and MC analyzed the data. CY, MC, and N edited the manuscript. CD and JK help in data collection and field coordination during the study. All authors contributed to the article and approved the submitted version.

## Funding

This study has been funded by National Health Mission, Chhattisgarh.

## Conflict of Interest

The authors declare that the research was conducted in the absence of any commercial or financial relationships that could be construed as a potential conflict of interest.

## Publisher's Note

All claims expressed in this article are solely those of the authors and do not necessarily represent those of their affiliated organizations, or those of the publisher, the editors and the reviewers. Any product that may be evaluated in this article, or claim that may be made by its manufacturer, is not guaranteed or endorsed by the publisher.

## Abbreviations

ASHA: Accredited Social Health Activist
CHW: community health workers
KAP: knowledge attitude and practices
LLIN: long lasting insecticidal nets
RDT: rapid diagnostic tests
WHO: World Health Organisation.

## References

[1] NVBDCP. National Framework for Malaria Elimination in India (2016–2030). (2016). Delhi: NVBDCP.

[2] Ranjha R Sharma A. Forest malaria: the prevailing obstacle for malaria control and elimination in India. BMJ Glob Health. (2021) 6:e005391. 10.1136/bmjgh-2021-005391 33990358 PMC8127975

[3] Mavalankar D. Doctors for tribal areas: issues and solutions. Indian J Community Med. (2016) 41:172–6. 10.4103/0970-0218.183587 27385868 PMC4919928

[4] Chourasia PK Verma A Pundir P Shukla N Chourasia MK. Underlying challenges in the path of malaria elimination: from India perspective. South Asian J Parasitol. (2020) 4:9–12. 10.24321/0019.5138.201901

[5] Chipukuma HM Halwiindi H Zulu JM Azizi SC Jacobs C. Evaluating fidelity of community health worker roles in malaria prevention and control programs in Livingstone District, Zambia—a bottleneck analysis. BMC Health Serv Res. (2020) 20:612. 10.1186/s12913-020-05458-1 32615960 PMC7331272

[6] WHO. Community Health Workers: What Do We Know About Them? (2007). Evidence and Information for Policy, Department of Human Resources for Health. Geneva: World Health Organization

[7] DOHFW CG. Mitanin Programme in Chhattisgarh, India (2021).

[8] NVBDCP. Available online at: https://nvbdcp.gov.in/index4.php?lang=1&level=0&linkid=420&lid=3699 (accessed November 18, 2021).

[9] Ranjha R. A knowledge, attitude and practices survey and entomological situation analysis in malaria endemic tribal villages of Surajpur District, Chhattisgarh, India. J Commun Dis. (2019) 51:1–5.

[10] Canavati SE Kelly GC Quintero CE Vo TH Tran LK Ohrt C. Risk factor assessment for clinical malaria among forest-goers in a pre-elimination setting in Phu Yen Province, Vietnam. Malar J. (2019) 18:435. 10.1186/s12936-019-3068-4 31861988 PMC6923829

[11] Ramalingareddy K. Improving health services for tribal populations. Int J Res Social Sci. (2016) 6:345–57.

[12] Gopalan SS Mohanty S Das A. Assessing community health workers' performance motivation: a mixed-methods approach on India's Accredited Social Health Activists (ASHA) programme. BMJ Open. (2012) 2:1557. 10.1136/bmjopen-2012-001557 23019208 PMC3488714

[13] Chourasia MK Raghavendra K Bhatt RM Swain DK Dutta GDP Kleinschmidt I. Involvement of Mitanins (female health volunteers) in active malaria surveillance, determinants and challenges in tribal populated malaria endemic villages of Chhattisgarh, India. BMC Public Health. (2017) 18:9. 10.1186/s12889-017-4565-4 28693465 PMC5504842

[14] Boakye MDS Owek CJ Oluoch E Atakora SB Wachira J Afrane YA. Needs assessment of community health workers to enhance efficient delivery of their services for community case management of malaria in Kenya. Malar J. (2021) 20:102. 10.1186/s12936-021-03640-2 33602242 PMC7891133

[15] Chowdhury P Baidya S Paul D Kalita B Saikia G Karmakar S. comparative study on knowledge and practice against malaria among Accredited Social Health Activists (ASHAs) of low and high endemic regions of Tripura, Northeast India. J Family Med Prim Care. (2020) 9:2420–5. 10.4103/jfmpc.jfmpc_1169_19 32754513 PMC7380810

[16] WHO. Global Technical Strategy for Malaria 2016–2030. (2015). Geneva: World Health Organisation.

[17] Rajvanshi H Nisar S Bharti PK Jayswar H Mishra AK Sharma RK. Significance of training, monitoring and assessment of malaria workers in achieving malaria elimination goal of Malaria Elimination Demonstration Project. Malar J. (2021) 20:27. 10.1186/s12936-020-03534-9 33413408 PMC7789890

[18] Ministry of Health and Family Welfare. Meeting People's Health Needs in Rural Areas: Framework for Implementation 2005–2012. National Rural Health Mission (2005).

[19] Diaz-Quijano FA Martinez-Vega RA Rodriguez-Morales AJ Rojas-Calero RA Luna-Gonzalez ML Diaz-Quijano RG. Association between the level of education and knowledge, attitudes and practices regarding dengue in the Caribbean region of Colombia. BMC Public Health. (2018) 18:143. 10.1186/s12889-018-5055-z 29338712 PMC5771071

[20] Crispin N Wamae A Ndirangu M Wamalwa D Wangalwa G Watako P. Effects of selected socio-demographic characteristics of community health workers on performance of home visits during pregnancy: a cross-sectional study in Busia District, Kenya. Glob J Health Sci. (2012) 4:78–90. 10.5539/gjhs.v4n5p78 22980380 PMC4776911

[21] Musoke D Ndejjo R Atusingwize E Mukama T Ssemugabo C Gibson L. Performance of community health workers and associated factors in a rural community in Wakiso district, Uganda. Afr Health Sci. (2019) 19:2784–97. 10.4314/ahs.v19i3.55 32127852 PMC7040253

[22] Sharma AK Aggarwal OP Chaturvedi S Bhasin SK. Is education a determinant of knowledge about malaria among Indian tribal population? J Commun Dis. (2003) 35:109–17. 15562957

[23] NVBDCP. Malaria Control Stratigies. (2021). Available online at: https://nvbdcp.gov.in/index4.php?lang=1&level=0&linkid=421&lid=3707 (accessed July 19, 2021).

[24] Roupa Z Polychronis G Latzourakis E Nikitara M Ghobrial S Chrysafi A. Assessment of knowledge and perceptions of health workers regarding COVID-19: a cross-sectional study from cyprus. J Community Health. (2021) 46:251–8. 10.1007/s10900-020-00949-y 33184744 PMC7660130

[25] Hemingway J Shretta R Wells TN Bell D Djimde AA Achee N. Tools and strategies for malaria control and elimination: what do we need to achieve a grand convergence in malaria? PLoS Biol. (2016) 14:e1002380. 10.1371/journal.pbio.1002380 26934361 PMC4774904

[26] Saprii L Richards E Kokho P Theobald S. Community health workers in rural India: analysing the opportunities and challenges Accredited Social Health Activists (ASHAs) face in realising their multiple roles. Hum Resour Health. (2015) 13:95. 10.1186/s12960-015-0094-3 26646109 PMC4673775

[27] Kawade A Gore M Lele P Chavan U Pinnock H Smith P. Interplaying role of healthcare activist and homemaker: a mixed-methods exploration of the workload of community health workers (Accredited Social Health Activists) in India. Hum Resour Health. (2021) 19:7. 10.1186/s12960-020-00546-z 33407518 PMC7789492

