# Supplementary material for: Knowledge Attitude and Practices of Mitanin's (Community Health Workers) in Chhattisgarh: Malaria Elimination Perspective
Source: Front Public Health. 2022 Mar 3;9:774864. doi: 10.3389/fpubh.2021.774864 (PMC8929527; doi:10.3389/fpubh.2021.774864)
Supplement: Supplementary file 1 [file Data_Sheet_1.docx]

**Supplementary data**

The following tables are results of ordinal regression for the individual components in KAP analysis. How individual component is affected by different dependent variables.

| **Supplementary Table 1: Factors affecting Mitanin’s Basic knowledge about malaria using ordinal logistic regression (n=203)** | | | | | | |
| --- | --- | --- | --- | --- | --- | --- |
| **Variable** | **Category** |  | **Univariate Analysis** | | **Multivariate analysis** | |
|  |  |  | **cOR (95%CI)** | **P value** | **aOR 95%CI)** | **P value** |
| Endemicity | Low | | 0.99(0.59-1.74) | **0.963** | 0.86(.44-1.68) | 0.669 |
|  | High | | 1 |  | 1 |  |
| Age | In years | | 0.99(0.96-1.02)) | 0.602 | 1.01(0.97-1.05) | 0.574 |
| Education | Less than primary | | 0.38(0.20-0.70) | **0.002** | 0.39(0.20-0.80) | **0.010** |
|  | Primary and more | | 1 |  | 1 |  |
| Experience | Less than 9 years | | 1.39(0.66-2.90) | **0.387** | 1.53(0.59-3.96) | 0.374 |
|  | 9-16 years | | 2.08(0.99-4.37) | **0.053** | 1.86(0.82-1.68) | **0.137** |
|  | 17 years and more | | 1 |  | 1 |  |
| P value < 0.05 statistically significant  Likelihood Ratio Chi-Square 11.10; 0.049 Pseudo R2 – 0.067 | | | | | | |

| **Supplementary Table 2: Factors affecting Mitanin’s knowledge about malaria prevention using ordinal logistic regression (n=203)** | | | | | | |
| --- | --- | --- | --- | --- | --- | --- |
| **Variable** | **Category** |  | **Univariate Analysis** | | **Multivariate analysis** | |
|  |  |  | **cOR (95%CI)** | **P value** | **aOR 95%CI)** | **P value** |
| Endemicity | Low | | 0.37(0021-0.63) | **<0.001** | 0031(0.17-0.60) | **0.000** |
|  | High | | 1 |  | 1 |  |
| Age | In Years | | 0.98(0.95-1.00) | 0.098 | 0.98(0.95-1.02) | 0.319 |
| Education | Less than primary | | 0.78(0.46-1.33) | **0.36** | 0.61(0.32-1.16) | **0.133** |
|  | Primary and more | | 1 |  | 1 |  |
| Experience | Less than 9 years | | 1.54(0.77-3.06) | **0.218** | 0.70(0.30-1.67) | 0.423 |
|  | 9-16 years | | 2.36(1.19-4.67) | **0.013** | 1.40(0.67-2.95) | 0.370 |
|  | 17 years and more | | 1 |  | 1 |  |
| P value < 0.05 statistically significant  Likelihood Ratio Chi-Square 21.3; 0.001 Pseudo R2 – 0.119 | | | | | | |

| **Supplementary Table 3: Factors affecting Mitanin’s Malaria treatment KAP Performance using ordinal logistic regression (n=203)** | | | | | | |
| --- | --- | --- | --- | --- | --- | --- |
| **Variable** | **Category** |  | **Univariate Analysis** | | **Multivariate analysis** | |
|  |  |  | **cOR (95%CI)** | **P value** | **aOR 95%CI)** | **P value** |
| Endemicity | Low | | 0.352(0.21-0.60) | **0.001** | 0.370.19-0.69) | **0.002** |
|  | High | | 1 |  | 1 |  |
| Age | In years | | 0.94(0.91-0.96) | 0.000 | 0.96(0.93-1.00) | 0.041 |
| Education | Less than primary | | 0.52(0.30-0.89) | **0.010** | 0.51(0.27-0.96) | **0.038** |
|  | Primary and more | | 1 |  | 1 |  |
| Experience | Less than 9 years | | 4.46(2.23-8.94) | **0.000** | 1.82(0.77-4.31) | 0.171 |
|  | 9-16 years | | 4.47(2.24-8.90) | **0.000** | 2.43(1.15-5.12) | 0.019 |
|  | 17 years and more | | 1 |  | 1 |  |
| P value < 0.05 statistically significant  Likelihood Ratio Chi-Square 42.12; <0.001 Pseudo R2 – 0.218 | | | | | | |
